# Supplementary material for: Are carbon emissions trading and green financial instruments synergistic? -Comprehensive quantitative research based on content analysis
Source: PLoS One. 2024 Mar 7;19(3):e0298601. doi: 10.1371/journal.pone.0298601 (PMC10919601; doi:10.1371/journal.pone.0298601)
Supplement: S1 Data — (ZIP) [file pone.0298601.s001.zip › Data/地级市Z_F工作报告.zip!/─■╧─/╬Γ╓╥╩╨/└┤╫╘╣1⁄2╓┌║┼í╛┬φ┐╦╩2╛▌═°í┐_2007.docx]

各位代表：

现在，我代表市人民政府，向大会作政府工作报告，请予审议，并请市政协各位委员和其他列席人员提出意见。

一、2006年工作回顾

2006年，各级政府和全市人民在自治区党委、政府和市委的正确领导下，以邓小平理论和“三个代表”重要思想为指导，以科学发展观统领全局，深入贯彻落实党的十六届五中、六中全会精神，励精图治，开拓创新，圆满完成了市二届三次人代会确定的各项目标任务，实现了“十一五”的良好开局。

——综合实力不断增强。预计全市完成地区生产总值116亿元，增长13.7%，连续三年高于全区平均水平。地方财政收入达到历史最好水平，全市完成8.55亿元，增长50%，其中市区、青铜峡、盐池、同心、红寺堡分别完成3.48亿元、3.05亿元、9120万元、4696万元和6403万元，增长61%、31%、61%、17%和167%。节能降耗和资源利用有了积极变化，万元GDP综合能耗预计降低5%。市域经济自主增长的活力显著增强，全市非公有制经济完成增加值41亿元，占GDP的35.3%。人居环境和人民生活水平进一步改善，全年城镇居民人均可支配收入8394元，农民人均纯收入3137元，分别增长11.7%和8.5%。在中国城市论坛组委会、世界银行等机构测评中，吴忠城市生活质量跃居全国50佳第41位，政府效率和投资环境综合排名列西北第一。青铜峡市跃居西部百强县第56位，被评为2006年度“全国最具投资潜力中小城市”百强，排名第76位。盐池、同心、红寺堡经济实力大幅提升。全市各行各业均呈现出快速发展、协调推进、欣欣向荣的新局面。

——工业经济提速增效。全市完成工业总产值199亿元、增加值55亿元，分别增长29.1%和27.9%。其中地方工业完成总产值80亿元、增加值24亿元，分别增长21%和20.5%。全市规模以上工业实现增加值49.3亿元、利润8.5亿元，分别增长27.6%和122%。工业拉动经济增长9.6个百分点，主导地位进一步凸现。冶金、化工、农副产品加工等优势产业不断壮大，骨干企业市场竞争力大幅增强。全市12家企业进入自治区重点工业骨干企业和非公有制工业骨干企业行列。投资500万元以上的新建、技改、扩建项目有55个开工建设，37个建成投产，完成投资20亿元。园区成为工业发展的重要平台。太阳山开发区资源勘探、基础设施建设、招商引资取得突破性进展，依法招标拍卖出让永安、韦一井田，完成韦二至韦五井田的煤炭资源详查，开工建设刘家沟水库、中盐高速公路连接线等26个项目，引进宁夏发电集团、庆华集团等大企业入园发展，形成大开发的可喜局面。金积工业园区顺利完成规划调整和资源整合，完成工业总产值18亿元。青铜峡工业区项目建设步伐加快，电力、冶金等五大主导产业产值达到133亿元，占该市工业总产值的92%。盐池东顺工业园区升格为自治区级开发区，入园企业达20家，完成工业总产值3.66亿元，占全县工业总产值的41.1%。同心羊绒工业园区组建了绒业集团有限公司，完成道路、给排水等基础设施项目建设，入园企业达34家。红寺堡工业园区招商引资和项目入园也取得明显成效。工业成为县域经济增长的主要推动力量。

——新农村建设扎实推进。全市完成农业总产值36.1亿元，增长7.5%。粮食生产能力稳步提高，总产量达到7.5亿公斤，增长9.5%；冬麦种植面积达到6.2万亩。特色农业种植规模不断扩大，全市酿酒葡萄、马铃薯、设施瓜菜、中药材面积分别达到7万亩、40万亩、5.6万亩和10.5万亩。畜牧业主导地位日渐突出，市区奶牛、青铜峡生猪和盐池滩羊、滩鸡等特色养殖业快速发展，全市奶牛饲养量13.9万头，新增2万头；鲜奶产量41万吨，增长18.2%；羊、生猪饲养量分别达到400.3万只、90.1万头，增长5.9%和34%。实现畜牧业产值16亿元，增长8.6%，占农业总产值的44%。市区被中国乳业协会评为“全国牛奶生产强县”，被国家食品药品监督管理局确定为“食品安全保证体系建设试点城市”。农业产业化进程加快，国家、自治区级农业产业化龙头企业达到17家，夏进乳业、万胜生物、御马酒业等企业辐射带动能力显著增强。农业标准化生产水平和农产品质量明显提高，获无公害农产品认证140个、产地认定11个。“三农”投入力度加大，农村基础设施不断改善，建成农村公路321.9公里。新修砌护各类渠道301公里。实施孙家滩引水工程。启动建设“塞上农民新居”示范点52个，农民新居建设和农村能源建设走在全区前列。新建农村户用沼气池5720座。市区金积镇北门村被确定为省部共建社会主义新农村示范村。实施天然林保护等林业建设工程，完成营造林面积31.2万亩。中部干旱带经济发展及抗旱救灾、扶贫开发工作扎实推进，市上投入100万元，盐池、同心、红寺堡分别筹集资金1965万元、517万元、152.4万元，帮助山区群众发展生产和解决人畜饮水困难，共建设集雨场8700多个，39个贫困村完成整村推进任务。同心人均劳务收入597元，红寺堡劳务收入占农民人均纯收入的40%以上。山区各县(区)在大旱之年经济发展、社会稳定、人民安居乐业，各项事业都有了新的发展。

——城乡建设步伐加快。全市安排城市建设项目230个，建筑面积166.7万平方米，完成投资22.5亿元。其中市区开工建设项目53个，建筑面积84.5万平方米，完成投资12亿元。全市城乡建设持续升温、日新月异。城市化率达到38%。滨河生态城市初具规模。完成滨河大道二期3.85公里主干线及3条连接线建设；新建续建金滨路等8条13.1公里城市道路，是近年来建设城市道路最长、投资规模最大、成效最明显的一年；吴忠黄河公路大桥项目建议书获国家发改委和交通部批复；实施城市集污及供水管网、供水供热分户改造工程，完成投资6000万元；新建续建华港佳苑等10个住宅小区，建筑面积62万平方米；开工建设城市医疗、卫生、学校等16个公共建筑项目，建筑面积13万平方米。城市园林绿化有了历史性的突破，实施滨河湿地恢复与保护、明珠公园续建等“一河三园八路”园林绿化工程，新增绿地面积35.6公顷。县城及中心集镇建设稳步推进，青铜峡加快城市道路建设和环境综合整治步伐，不断提升城市品位，入围中国特色魅力城市200强。盐池完成县城总体规划修编，新建改造城市道路6.5公里，实施盐州园等绿化工程，新增城市绿地595亩，县城绿化覆盖率达26.8%。同心按照“五路一园一桥一场”的建设思路，启动民间资金8500万元，新建改造西北环路等重点项目，完成投资2.65亿元。红寺堡新建、改建综合市场等一批项目，狠抓城市绿化、亮化、美化工作，“八路九街”城市框架全面拉开。金积、太阳山等中心集镇建设步伐加快，城镇功能不断提升，聚集效能不断增强。

——改革开放不断深化。企业产权制度改革成效显著，完成吴忠市印刷包装公司、青铜峡峡光纸业公司、盐池县汽车运输公司等企业改制，解决36家企业改制遗留问题，市区益木公司、瑞特纸业公司等企业进入破产改制程序。农村综合改革扎实推进，“两免一补”政策全面落实。财政管理、社会保障、医疗卫生、科技教育和文化体制改革稳步推行。组织参加香港宁夏活动周、西安投洽会和第18届西交会，成功举办青铜峡首届“中国县长论坛暨西部百强县县长峰会”、首届“中国宁夏(盐池)滩羊节”等活动，知名度和吸引力得到显著提升。广泛开展对外交流合作，市上先后与马来西亚、纳米比亚的一些城市建立友好合作关系，与湖南长沙、上海联交所进行密切合作，与宁夏大学开展科技和项目合作。各县（市、区）积极深化与发达地区的经济技术合作交流，广泛开展招商引资，加强区域合作，取得显著成效。全市引进项目110个，实际到位资金24.12亿元，增长12.2%；进出口企业达到36家，进出口总额9421万美元，是2005年的11倍。

——第三产业加快发展。全市完成社会消费品零售总额27亿元，增长11.6%，第三产业增加值占GDP的30.5%。深入开展“现代商贸流通经济发展年”活动。商贸核心区建设步伐加快，街心公园对外开放，吴忠宾馆、红宝宾馆投入运营，迎宾大街完成南段打通，明珠广场周边及迎宾大街商业氛围日渐浓厚。全面实施涝河桥市场区域改造工程，完成307县道过境段拓宽、广场建设等年度施工任务，有效提升了市场功能。农村流通体系不断完善，消费市场日趋活跃。“万村千乡”市场工程顺利实施，建成乡村骨干店185家。青铜峡积极推行“放心农资下乡进村”连锁经营试点，有力推动流通企业向镇、村及社区延伸。盐池中心商场、万家福购物广场建成开业。同心瀛海建材城、奇虎商厦等项目进展顺利。红寺堡大河、南川农贸市场开业运营。各县（市、区）城乡市场更加繁荣，商贸流通更加活跃。挖掘整合旅游资源，培育发展特色旅游业，率先在全区开展旅游行业“争星创优”活动，举办宁夏吴忠第二届旅游产品博览会、青铜峡第三届黄河旅游文化节、盐池第四届文化旅游节。盐池、同心进入全国百家红色旅游经典景区行列。

——社会事业全面进步。公安科技创新工作不断加强，我市被公安部、科技部确定为全国第二批科技强警示范城市，成为西北地区唯一参与创建的非省会城市；市区率先在全区实现教育强区、强乡镇目标，青铜峡启动教育强市强镇工作，山区全面实现“两基”攻坚目标。吴忠职业技术学院人才培养工作通过教育部评估，并更名为宁夏民族职业技术学院。率先在全区开展农村面源污染综合整治工程。医疗服务、医药监管和疾病预防控制体系不断完善，市区、青铜峡及盐池全面启动新型农村合作医疗。完成红寺堡人民医院等8个市(县)医院、20个乡镇卫生院、379个标准化村卫生室新建扩建项目，基层医疗卫生条件不断改善。全年新增城镇就业12483人，其中下岗失业人员再就业1789人，城镇登记失业率为4.05%。城镇职工养老、医疗、失业、工伤、生育保险参保人数突破20万人。完善城市和农村低保救助体系，救助受灾群众13.9万人，开展城乡医疗、教育、住房救助7800多人次。投资1200万元，完成市区三个乡镇安全饮水工程，解决5万农民饮水安全问题。市区5家农林牧场、3600多名职工全员进入养老保险工作有了新进展。14家国有企业1815名职工经济补偿和38家国有关闭破产、困难企业3907名退休人员的医疗保险问题已纳入自治区解决范围。成功举办建党85周年歌咏比赛、盐池解放70周年、豫海县回民自治政府成立70周年纪念活动和市区第五届农民运动会。盐池获“全国文物保护先进县”荣誉称号。开展“3+1”送文化下乡活动。广泛开展全民健身运动，组织参加全区第十二届运动会。开展计划生育“三无”、“一无”乡(镇)创建活动，推进“少生快富”工程和奖励扶助制度，人口自然增长率为10.11‰，计划生育率为90.94%。国土资源、环境保护、安全生产监管工作取得新成效，安全生产控制指标降幅居全区首位。组织防空袭警报和防震减灾模拟演练等活动，不断加强国防动员教育和人民防空工作。“双拥”模范城创建活动取得明显成效。深入贯彻落实党的民族宗教政策，平等、团结、互助的新型民族关系不断巩固和发展。统计、广播电视、物价、残联、住房公积金等社会事业有了新的发展。

——精神文明和民主法制建设得到加强。深入开展社会主义荣辱观教育活动，出版发行《吴忠与灵州》、《魅力吴忠》等系列丛书，拍摄完成《吴忠山河》宣传片。民主法制进程加快，深化审批制度改革，集中清理行政许可事项，推进行政管理体制创新，扩大政务公开范围。基层民主政治建设不断加强，社区居委会换届选举工作顺利进行。各级政府自觉接受人民代表大会及其常务委员会的依法监督和人民政协的民主监督，区、市及市区三级人大代表议案9件、建议意见122件和政协委员提案319件全部办复完毕。积极解决人民群众关注的热点难点问题。市人民政府承诺为民办理的22件实事基本落实。其中：新建农村户用沼气示范户1500户；加强食品安全，设立无公害蔬菜专销点3个；新增市区城市绿地30公顷，新绿化具有园林景观的城市道路3条等实事提前完成。不断强化审计监督和行政效能监察，深入开展治理商业贿赂工作，扎实推进整顿和规范市场经济秩序。高度重视和加强信访工作，坚持和完善市级领导信访接待日制度，切实解决人民群众反映的实际问题。全面开展“平安吴忠”创建活动，启动“五五”普法，法律援助工作进一步加强，被国家司法部、残联授予“维护司法公正奖”。盐池获“全国法制宣传教育先进县”称号。加强社会治安综合治理，严厉打击各种违法犯罪活动，人民群众的安全感进一步增强。

各位代表，回顾过去的一年，我们欣慰地看到，经过各族人民的共同努力，全市经济更加发展，政治更加民主，社会更加进步，人民更加富裕，向构建和谐社会的目标迈出了坚实的一步，增强了奋勇争先、加快发展的激情和魄力。这些成绩表明，各级政府和部门面对新的困难和挑战，认真贯彻落实科学发展观，在认识上有了新提高，在实践上有了新进展，在工作上有了新突破。这是自治区党委、政府亲切关怀和市委正确领导的结果，是市人大、市政协及社会各界有效监督和大力支持的结果，是全市人民团结奋进、无私奉献的结果。在此，我代表市人民政府，向全市各族人民，向驻吴部队、武警官兵、公安干警、中央及区属驻吴单位，向各位人大代表、政协委员、各民主党派、工商联、无党派人士、各人民团体以及关心、支持吴忠发展的离退休老同志和社会各界人士，表示崇高的敬意和衷心的感谢！

认真总结过去的工作，我们也清醒地看到，全市经济社会发展中还存在不少困难和问题。主要是：市域经济增长方式较为粗放，资源、能源消耗较高，发展的质量和效益水平还不高；高耗能企业节能改造、造纸企业污水治理成效欠佳，节能减排、环境保护压力较大；优秀企业经营管理人才短缺，企业自主创新能力不强，做大做强的魄力不大；农村基础设施建设投入不足，农业结构调整强度不够，山区抗旱和扶贫开发任务比较艰巨，部分群众生产生活困难，新农村建设面临一些新问题；全社会固定资产投资增幅不高，新开工建设的大项目较少，城市建设融资难度加大，重点项目建设欠账较多；促进财政收入稳定增长的新财源较少，财政基础薄弱，刚性支出压力大，可用财力少；就业、社会保障压力加大，困难群众生活救助、失地农民转移就业等一些关系民生的问题尚未得到有效解决；投资环境还不理想，招商引资质量不高，资金到位率低；有的政府部门服务水平、办事效率还不高等。对此，我们要不断探索，勇于实践，认真加以解决。

二、2007年工作任务

2007年是实施“十一五”规划的关键之年。我市加快经济社会发展既有许多有利因素，也面临着多方面的挑战和压力。世界经济逐步回升，结构调整步伐加快，国家继续实施稳健的财政政策和货币政策，继续加大对西部地区的倾斜和支持力度，机遇稍纵即逝。当前，周边地区争先发展的浪潮汹涌激荡，竞争形势催人奋进，发展犹如逆水行舟，不进则退。我们必须以只争朝夕、奋力赶超的勇气，认真学习借鉴山东、河南等地区大手笔、大气魄的发展理念和创业精神，将大挑战转化为大机遇，想大事、谋大事、干大事，敢为人先，勇往直前，努力在新一轮区域竞争中争先进位。

按照市委三届二次全体(扩大)会议精神，2007年政府工作的总体要求是：深入贯彻落实党的十六届五中、六中全会精神，牢固树立和落实科学发展观，按照构建社会主义和谐社会、全面建设小康社会和推进社会主义新农村建设的要求，解放思想，创新管理，优化环境，深化改革，扩大开放，着力做强工业，做精农业，做美城市，做活商贸，统筹城乡和社会发展，关注和解决民生问题，努力推进经济社会又好又快发展，为建设小康吴忠、实力吴忠、魅力吴忠、和谐吴忠夯实基础。主要预期目标和约束性指标是：地区生产总值完成130亿元，增长12%；全社会固定资产投资完成65亿元，增长15%；地方财政收入达到7.38亿元，可比增长13%，其中市区3.05亿元，可比增长13%；城镇居民人均可支配收入达到9000元，增长7%，农民人均纯收入达到3320元，增长6%。城镇登记失业率控制在4.5%以内；人口自然增长率控制在11.5‰以内；万元GDP综合能耗降低4%，主要污染物总量减排3%。实现上述目标，必须从全局上坚持和把握好以下四个关键：

一是坚持科学发展。以科学发展观统领全局，切实转变增长方式，推进经济结构调整，更加注重环境保护和节能降耗，大力发展循环经济，推进节约发展、清洁发展和安全发展，加快城镇化、信息化和新型工业化进程，科学务实地建设社会主义新农村，努力实现速度、质量、效益相协调，消费、投资、出口相协调，人口、资源、环境相协调，真正做到又好又快发展。

二是坚持和谐惠民。始终把发展经济与造福群众统一起来，把经济建设与社会进步协调起来，把川区加快发展与山区脱贫致富兼顾起来，把推进城市化进程与解决好失地农民生产生活问题结合起来，以人为本，关注民生，更加积极地正视问题、化解矛盾，最大限度地增加和谐因素，集中民智，协调推进，不断促进社会和谐进步。

三是坚持改革创新。把改革创新贯穿到工作的各个环节，鼓励全社会，特别是企业等市场主体，勇于改革，大胆创新，务求在机制创新、管理创新和科技创新等改革的重点领域、发展的关键环节、政府的公共服务、企业的专有技术上取得新突破、新进展，以改革破解发展中的难题，以创新激发全社会的活力，形成百姓创家业、能人创企业、干部创事业的浓厚氛围。

四是坚持项目带动。抓住国家支持民族地区和欠发达地区农业、服务业、社会事业、节能环保等发展的机遇，积极争取项目和资金支持。进一步深化对外开放和合作，提高开放合作水平，营造有利的发展环境。鼓励和支持部门、企业及社会力量对外开展经济技术合作，大力推进招商引资，突出引大引强、靠大靠强，争取引进新上一批大项目、大企业，为加快经济发展增添后劲。

重点做好以下六个方面的工作：

(一)强力推进新型工业化，加快工业结构优化升级

按照“打造工业园区，壮大优势产业，做强龙头企业，发展循环经济”的思路，依托信息技术，加快结构调整，转变增长方式，推进新型工业化。全市工业总产值、规模以上工业增加值和利税增幅均在15%以上。突出抓好工业园区建设。科学规划园区基础设施和产业布局，完善运行机制和政策，加快资源开发利用，打造工业发展平台。太阳山开发区年内完成供水工程、中盐高速公路连接线等项目，积极做好韦二、韦三井田的资源出让和招商工作，抓好庆华循环经济工业园等6个产业园区的规划建设。金积工业园区加快造纸化工循环经济园道路、供电、给排水等基础设施建设，做好2×30万千瓦热电联产项目、第二污水处理厂等前期准备工作。加快青铜峡工业区、盐池东顺工业园区、同心羊绒产业园区、红寺堡工业园区建设，力争使更多的大项目入驻园区，提高园区核心竞争能力。加快培植优势特色产业集群。加快培育太阳山煤炭开采、煤化工、综合发电、金属镁等六大产业集群，逐步建成具有区域性示范作用的工业园区。抓好大塘大坝发电公司2×60万千瓦发电机组、青铝集团2×30万千瓦自备电厂和红寺堡风电厂等5个5万千瓦风力发电项目建设，壮大能源电力产业。抓好庆华集团年产100万吨煤焦化、蒙欣公司年产60万吨兰碳、宁鲁石化120万吨重油催裂化等项目，做大冶金化工产业。依托昊盛纸业公司，规划整合市区造纸产业，搬迁新源纸业公司，开工建设日处理150吨黑液碱回收项目，实现集中治污，改造提升造纸产业。力促万胜生物公司年产3.3万吨谷氨酸项目投产达效，加快吴忠卷烟厂5万箱生产线技改扩建、祥福绒业精纺羊绒纱、双维绒业牦牛绒面料等项目建设，做强农副产品加工业，力争年内完成投资20亿元，新增产值18亿元，努力把工业园区打造成为承接产业转移的基地、推进集群发展的载体和扩大对外合作的平台。大力推动循环经济发展。切实抓好金昱元、万胜生物、昌鑫建材循环经济试点。依托金牛化肥公司扩大硫酸钾、电石产能，规划建设盐酸、PVC树脂等项目，逐步形成产值10亿元的化工循环经济产业链。积极推进节能降耗，加强对化工、造纸、建材等行业和重点企业的节能管理，提高资源利用率。加快改造高耗能产业，加大资产重组力度，改进关键技术，淘汰落后工艺和设备，不断推动企业降低能耗、提高效益。

(二)统筹“三农”发展，推进社会主义新农村建设

紧紧围绕农业稳定发展、农民持续增收、农村更加和谐这一主题，调整优化农业结构，发展现代农业，改善农村基础设施，加快社会主义新农村建设步伐。提高农业综合生产能力。认真落实粮食、化肥、良种、农机具、机修基本农田等五项直补政策，继续加大对农业和农村的投入力度。切实保护耕地，稳定粮食种植面积，发展优质粮食产业，全市粮食播种面积保持在300万亩，其中优质粮食100万亩，冬麦10万亩，总产稳定在7亿公斤以上。实施太阳山土地开发、青铜峡灌区唐徕渠和西干渠合并工程，盐池、同心、红寺堡境内小流域综合治理工程，改造中低产田8万亩，新增灌溉面积3万亩。实施退耕还林草、封山禁牧工程,完成退耕还林面积4万亩，新增封育面积8万亩，续封6万亩。大力发展现代农业。突出抓好吴忠国家农业科技园区建设，实施精准农业技术研究国际合作项目，示范推广蔬菜无公害化和奶牛健康养殖新技术，创新运行机制和发展模式，使之成为全市现代农业的示范区。积极应对奶业市场变化，强化良种繁育、疫病诊疗和信息管理体系建设；继续实施“奶牛出户入园上挤奶台”工程，推进金银滩优质奶牛核心区建设，年内奶牛饲养量达到17万头，新增3.1万头。保护盐池滩羊品牌和种质资源，滩羊饲养量达到135万只。依托“中国甘草之乡”品牌，人工种植甘草累计达到15万亩。加快发展酿酒葡萄产业，年内新增4万亩，达到11万亩。青铜峡农作物及蔬菜制种面积达到7.5万亩。实施龙头企业振兴工程，支持茂源公司实施果汁生产线扩建项目，带动高酸苹果产业加快发展，种植面积达到4万亩。依托涝河桥、沙漠王子和盐池荣宝园等品牌和企业，促进牛羊肉深加工增值。加强农村基础设施建设。加快农村公路建设步伐，新建、改造农村公路200公里。实施农村安全饮水工程，解决6万人口安全饮水问题。实施生态家园项目，加快改水、改厕、改灶等农村小康环保行动计划步伐，建设“一池三改”户用沼气池9710座。实施“千村整治”工程，加快乡村供排水、村庄绿化、清洁能源等建设，改善农村人居环境。开展村企互动、共建新农村活动。切实抓好川区40%以上、山区30%以上的村庄建设规划，实施山区危房危窑改造项目，新建续建20个“塞上农民新居”和村庄整治示范点。加快山区扶贫攻坚步伐。切实加强中部干旱带抗旱节水等基础设施建设，实施贫困村整村推进计划，抓好生态移民工程。加快盐池、同心旱作节水农业科技示范园区、固海扩灌区农田配套等项目建设，发展节水农业。推进韦州、下马关灌区旱改水易地生态移民、孙家滩移民开发工程建设进度。实施同心东部引水扩灌、窑山引水、盐池石山子、杨二庄供水工程，切实解决山区人畜饮水困难。实施“百万农民培训工程”、“阳光工程”，提高农民致富创业本领。大力发展劳务经济，全年输出劳务稳定在22万人以上。

(三)加大城乡建设力度，积极推进城镇化进程

紧紧抓住自治区建设沿黄城市带的机遇，运用市场机制，加快滨河生态城市建设步伐，提升县城和中心集镇的发展水平，全面推进城镇化进程。全市新建续建城市建设项目120个，建筑面积140万平方米，投资18.7亿元。提升滨河生态城市功能。加快市区城市西移和近水亲河，集中规划实施一批重点基础设施项目。开工建设吴忠黄河公路大桥。实施罗家湖至细腰子土拜10.6公里的滨河大道三期工程。新建扩建金滨路北段等6条城市道路，启动建设秦渠以南6平方公里集污排水项目，实施城市集中供热扩建、供热分户控制改造工程，开工建设新区汽车站、医疗垃圾废弃物集中处理等6个公共建筑项目，加快锦秀园等6个住宅小区建设，建筑面积60万平方米。配合实施中盐高速公路、中银太铁路过境段和古窑子至青铜峡高速公路等项目。强化城市生态环境建设。实施10万亩城市林业建设整治工程，推进市区滨河湿地、盐池和同心城市防护林、青铜峡工业区和太阳山开发区生态治理等项目，新建城市景观林、城市防护林、城市经济林7.1万亩，保护城市湿地1.9万亩，恢复湖面1.2万亩。实施罗家湖、明珠公园续建、西一环南北段等“一湖二园六路”园林绿化工程，市区新增绿地面积40公顷。抓好县城及中心集镇建设。重点实施青铜峡黄河西岸滨河大道和汉延渠景观绿化，盐池环城绿化和瓮城古迹修复，同心北沟生态环境治理、县城环境综合整治，红寺堡集中供热和生活垃圾处理等工程。抓好金积、金银滩、峡口、惠安堡、韦州、太阳山等中心集镇的规划和建设，建成一批风格独特、聚集产业的小城镇，提升城镇辐射带动功能。

(四)培育壮大商贸流通业，提升第三产业发展水平

按照“完善市场功能，整合商贸资源，发挥商贸优势，打造商贸名城”的思路，加快完善政策扶持体系，建立商贸流通业发展基金，制定项目扶持和资金补助办法，落实税费优惠政策，鼓励商业银行扩大信贷规模，支持商贸流通企业完善基础设施，培植壮大现代商贸流通经济，带动第三产业发展，推进商贸活市。年内全社会消费品零售总额达到30亿元，增长12%。加快发展现代商贸流通业。开工建设吴忠国际商贸大厦，改造整合明珠广场周边商业环境和资源，加快建设商贸核心区。培育发展特色商业街区，规划实施西部物流中心项目。积极抓好青铜峡星级宾馆、鑫田购物城、物流中心，同心建材、农产品批发市场，红寺堡专业市场等项目，构建遍布城乡、辐射周边的商贸流通网络。大力发展社区服务、电子商务、物流配送、中介服务、信息、保险等现代服务业。加快建立和完善农村流通体系，协调推进“名店下乡”工程、“万村千乡”市场工程，引导和鼓励有实力的商贸企业到农村发展，扩大农村消费，繁荣农村市场。加快发展特色旅游业。以青铜峡黄河大峡谷旅游区为龙头，加快整合鸟岛、金沙湾、108塔、董府、滨河湿地、古城湾等沿黄带的旅游资源，彰显塞上江南魅力；进一步开发盐池、同心、红寺堡地区的旅游资源，培育新的旅游景点，把盐池革命烈士纪念馆、同心红军西征纪念园建成国内有影响的红色旅游经典景区。

(五)提升协调发展水平，促进和谐社会建设

按照统筹经济与社会发展的思路，在加快经济建设的同时，协调推进各项社会事业，促进经济社会可持续发展和人的全面进步。推动科技创新和优先发展教育。切实加强科技创新工作，深化和高等院校、科研院所的技术交流与合作，加快工业新技术及农业新品种的开发应用。深入开展科技特派员创业行动，有选择地在重点领域实施重大科技项目，提高科技对经济增长的贡献率。深化教育强县(区)建设，完成青铜峡教育强乡镇创建工作。巩固提高“两基”水平，确保高标准通过国家“两基”复查验收。大力实施素质教育，市区城区普及高中阶段教育。坚持公共教育资源向农村、贫困地区倾斜，缩小城乡、区域教育发展差距。认真落实义务教育阶段免除学杂费和“两免一补”政策，促进山川教育均衡协调发展。加强民族教育和职业技术教育，提升宁夏民族职业技术学院教育质量和办学水平，积极争取3所中小学进入全区“百标学校”行列。抓好市区巴浪湖学校等中小学改扩建项目，确保年内建成投入使用。认真组织好大中专毕业生就业推介，加快企业管理、专业技术人才的引进、培养，壮大人才队伍。加快发展公共卫生和文体事业。继续深化医疗卫生体制改革，加强农村三级卫生服务网络和以社区卫生服务为基础的新型城市卫生服务体系建设，建立覆盖城乡居民的基本卫生保健制度。加强艾滋病等传染病的监控与防治工作。全面推进新型农村合作医疗，提高参合农民受益水平和覆盖范围。积极推进乡镇卫生院人事制度改革。加大医疗市场监管力度，整顿和规范医疗市场。深入开展社会主义核心价值体系、社会主义荣辱观和法制理念教育活动，开展文化扶贫、“三下乡”、“四进社区”等群众性精神文明创建活动，提高全社会的文明程度。加强公益性文化设施建设，规划建设吴忠图书馆、万人体育场等项目，突出抓好社区和乡镇综合文化站(室)、文化信息资源共享工程建设。继续开展“扫黄打非”行动，净化文化市场。深入开展全民健身运动，举办市直机关第二届运动会。大力整顿和规范市场经济秩序，依法整治危害食品安全、从事传销、制售假冒伪劣产品等行为。加强环境保护和人口计划生育工作。实施和完善环境发展综合决策考核机制，严格落实环境影响评价第一审批制度，强化企业激励和约束机制，促使企业加强技术改造，实行清洁生产。继续开展计划生育“三无”、“一无”乡(镇)创建工作，扎实推进“少生快富”工程和农村部分计划生育家庭奖励扶助制度，依法控制山区人口增长，稳定川区低生育水平。同时，深入贯彻党的民族宗教政策，切实做好新时期的民族宗教工作。加强国防动员、人民防空和民兵预备役建设，创建“全国双拥模范城”。认真做好全国第二次农业普查工作。加快气象防灾减灾体系建设。加强财税征管工作，深化财政体制改革，加快公共财政建设，推进“乡财县管”改革，启动化解乡镇债务工作。加强工商、技术监督、通信、金融、供电等工作，进一步提高推进发展、构建和谐的能力。

(六)以人为本，关注民生，切实维护人民群众的根本利益

从解决人民群众最现实、最关心、最直接的问题入手，促进社会公平，维护社会稳定。认真做好就业再就业工作。进一步加大税费减免、小额贷款等再就业扶持政策的落实力度，鼓励支持企业吸纳下岗失业人员。积极开展再就业援助活动，最大限度地帮助“4050”人员、失地农民、困难群体和残疾人实现再就业。实施城乡特困家庭培训就业援助工程，培训城乡特困家庭学员1500人。着力帮助零就业家庭和就业困难人员就业。全年城镇新增就业1万人，下岗失业人员再就业1000人，城镇各类人员职业技能培训4500人。加快完善社会保障体系。进一步完善城镇职工基本养老保险、基本医疗保险和失业保险制度，提高生育保险覆盖面，强化社会保险扩面征缴，力争全年养老、医疗、失业、工伤、生育保险人数突破21万人，基金征缴率达到98%以上。健全和完善城市居民最低生活保障及救助制度，开展向特困职工送温暖活动，做到动态管理下的应保尽保。提高农村“五保”人员集中供养率，全面推进农村大病医疗救助工作，完善农村特困群体救助制度，妥善解决困难群众和农民工子女上学、看病就医等方面的实际问题。全力维护社会稳定。全面落实安全生产责任制，加大安全生产投入，加快消防体系建设，增强企业防范重特大事故的能力。加强食品安全工作。完善信访工作机制，做好矛盾纠纷排查和调解工作，不断提高应对处置各类突发事件的能力。加强“五五”普法工作，深入开展基层依法治理示范单位创建活动。加强社会治安防控体系建设，依法严厉打击各种刑事犯罪和黑恶势力，保障人民安居乐业。

三、全面推进政府自身建设和管理创新

积极适应发展市场经济和构建和谐社会的需要，加快推进政府自身建设和管理创新，努力建设服务政府、法治政府、责任政府和效能政府，为人民服务、对人民负责、受人民监督、让人民满意。

转变职能，努力建设服务政府。深化行政审批制度改革，继续集中清理行政许可事项，明确审批权限，简化和规范审批程序。扩大进驻政务大厅窗口服务部门和事项，完善“一站式”服务机制，提高行政审批效率。加快电子政务建设。提升农村医疗保险、安全生产、财政管理、社会保障等社会管理和公共服务职能。建立和完善重大消防、自然灾害、供电供水、通信交通、防震减灾等突发事件应急机制。加强政务诚信、商务诚信、社会诚信建设，为各类投资主体提供宽松的发展环境。

依法行政，努力建设法治政府。强化法制理念，转变行政执法方式，规范行政执法行为，形成权责明确、行为规范、监督有效、保障有力的行政执法体制。充分发挥政府法律顾问团在行政管理事务中的作用。拓宽市长专线、社情民意调查等渠道，建立社会舆情汇集分析体系。坚持重大事项、重要情况请示报告制度和部门决策问责制度，完善部门绩效评估指标体系。健全公开听证制度，完善公众参与、专家论证和政府决策相结合的行政运行体制，提高政府执行力和公信力。

强化监督，努力建设责任政府。认真执行人民代表大会及其常务委员会的决定决议，全面支持人民政协发挥政治协商、民主监督和参政议政作用，积极办理人民代表议案、建议、意见和政协委员提案。密切与各民主党派、人民团体和社会各界的联系，不断拓宽社情民意反映渠道。定期召开与人大、政协、工会的联席会议，加强与社会各界的沟通和联系，力争使各项决策更加科学、符合实际。抓好关键环节的监督，拓宽监督渠道，增强监督的合力和实效。加强行政监察，推行“阳光行政”，坚决纠正部门和行业不正之风，促进政风行风的进一步好转。

改进作风，努力建设效能政府。深入贯彻中纪委七次全会精神，认真落实党风廉政建设目标责任制，健全权力运行监督约束机制。深入开展治理商业贿赂专项行动，加大对全市工程建设、产权交易、土地出让、医药购销、政府采购等重点领域的监管力度。深入开展“机关作风建设年”活动，全面加强各级干部思想、工作、生活作风和学风建设，讲操守，重品行，培养健康的生活情趣，保持高尚的精神追求。大兴求真务实之风，多作一些深入实际的调查研究，多解决一些影响改革发展稳定的突出问题，多办一些为人民群众排忧解难的实事，促进工作作风大转变。加强和改善公务员教育管理，严格考核奖惩制度，建设一支政治坚定、业务精通、作风过硬、人民满意的公务员队伍。

各位代表，和谐凝聚力量，和谐成就事业。全市各族人民正满怀信心在全面建设小康社会、构建社会主义和谐社会的伟大征途中阔步前进。让我们紧密团结在以胡锦涛同志为总书记的党中央周围，高举邓小平理论和“三个代表”重要思想伟大旗帜，在自治区党委、政府和市委的坚强领导下，紧紧依靠全市回汉各族人民，同心同德，再接再厉，求真务实，锐意进取，全力推进我市经济社会又好又快发展，为建设富裕文明和谐的新吴忠而努力奋斗！
